# Design of 2D Planar Sparse Binned Arrays Based on the Coarray Analysis

**DOI:** 10.3390/s21238018

**Published:** 2021-11-30

**Authors:** Óscar Martínez-Graullera, Júlio Cesar Eduardo de Souza, Montserrat Parrilla Romero, Ricardo Tokio Higuti

**Affiliations:** 1 Instituto de Tecnologías Físicas y de la Información (ITEFI-CSIC), C/Serrano 144, 28006 Madrid, Spain; m.parrilla@csic.es; 2 Faculdade de Engenharia, Universidade Estadual Paulista (UNESP), Campus Ilha Solteira, Avenida Brasil, 56, Ilha Solteira 15385-000, SP, Brazil; julio.c.souza@unesp.br (J.C.E.d.S.); ricardo.t.higuti@unesp.br (R.T.H.)

**Keywords:** beamforming, sparse arrays, ultrasonic imaging

## Abstract

The analysis of the beampattern is the base of sparse arrays design process. However, in the case of bidimensional arrays, this analysis has a high computational cost, turning the design process into a long and complex task. If the imaging system development is considered a holistic process, the aperture is a sampling grid that must be considered in the spatial domain through the coarray structure. Here, we propose to guide the aperture design process using statistical parameters of the distribution of the weights in the coarray. We have studied three designs of sparse matrix binned arrays with different sparseness degrees. Our results prove that there is a relationship between these parameters and the beampattern, which is valuable and improves the array design process. The proposed methodology reduces the computational cost up to 58 times with respect to the conventional fitness function based on the beampattern analysis.

## 1. Introduction

Progress in ultrasonic array manufacturing has allowed the commercialization of highly-populated apertures [1,2]. However, these high-density transducers are difficult to use due to limitations imposed by data acquisition systems. A large number of transducers introduces complexity to the imaging system, demanding the same number of electronic channels and generating a considerable volume of data.

Although it is possible to manufacture two-dimensional matrix arrays with more than a thousand elements, off-the-shelf commercial equipments are limited to hundreds of electronic channels. To address this mismatch, one proposed solution is to perform analogue prebeamforming, adapt the number of transducers to the number of electronic channels and use a conventional hardware system [3]. Despite the fact that this solution provides practical images of anatomical structures in medical applications, a significant part of the ultrasonic information is missing at the analogue processing stage.

On the other hand, there is an increasing interest in synthetic aperture imaging methods [2,4,5], where all signals corresponding to each pair of emission and reception elements in the array are independently acquired, and the focusing process is performed as a part of digital signal processing operations. These solutions can be implemented with a trade-off between parallel resources and acquisition time and, although there is lower SNR (Signal-to-Noise Ratio) when compared to phased-arrays, the complete set of signals is rich in information [6,7].

The capability of this analysis to provide new layers of information over conventional ultrasound images is an exciting field of study [8], with special interest for quantitative ultrasound medical diagnosis. However, in volumetric imaging, these techniques introduce challenges at several levels [9,10,11], including signal processing, system architecture, etc.

Sparse arrays were initially proposed as a manufacturing solution to reduce the number of active channels in high-density arrays [12,13]. Nowadays, they are a solution that allow the use of wide-aperture arrays with conventional hardware systems, and to perform synthetic aperture imaging with a reasonable cost. Mainly, optimization-based sparse array design strategies employ fitness functions (FF) based on the analysis of beampattern characteristics, such as mainlobe width and sidelobe level. Due to the high computational cost associated with the beampattern simulation and the complexity of its analysis [14,15], the use of these FFs turn the bidimensional sparse array design into a complex process [16,17,18,19].

From signal processing theory, the emission/reception array operation can be modelled by the coarray [20,21], which is formulated as part of the far-field narrowband analysis. This concept, obtained by the convolution of emission and reception apertures, constitutes a grid where each pair of emission-reception elements occupies a location in it as a sampling element. Although the coarray is not accurate to model directly the wideband pulse-echo response out of focus, it is used to study the spatial distribution of redundancy and is often used in the design of wideband systems [21,22,23]. The weight of each coarray element depends on the number of emitter/receiver pairs that are coincident at the same position, thus determining the redundancy of the sampling grid. The shape of the spatial distribution of coarray elements is defined here as natural apodization and determines the beampattern characteristics.

In sparse linear arrays, several works have addressed the array design process to obtain a uniform complete coarray [24,25,26]. For bidimensional arrays, in [23] a study of the behaviour of the coarray pattern generated by different combinations of emission and reception sub-apertures is presented. Moreover, previous to this work, several classical solutions like the Mills Cross array and the Vernier apertures were proposed to achieve this objective, albeit with limited success [22,27,28].

From this point of view, one objective of the sparse array design process is to improve the spatial information provided by the coarray. Therefore, instead of computing the beampattern, we base our efforts on the analysis of the statistical parameters of the coarray distribution. These statistical parameters are easily evaluated and have low computational cost.

In this paper, our objective is to propose a new fitness function which is efficient to predict the global beampattern behaviour, and which is computationally more efficient than the conventional analysis based on the measurement of specific beampattern characteristics. This work is developed in two stages. At first, the beampattern characteristics are studied, and a large set of random apertures is analyzed to evaluate which coarray distribution properties are relevant for the beampattern behaviour analysis. Second, two different fitness functions are proposed: the first one limited to the coarray parameters, and a second one where beampattern and the coarray parameters are combined, leading to more optimised results. To illustrate the effectiveness of the process, several solutions with different sparseness degrees have been studied for a specific array prototype. In this work, for simplicity and convenience with the design strategy, we have chosen to cope with the problem of sparse binned matrix array design [26].

## 2. Beampattern Analysis

### 2.1. Problem Description

In ultrasonic imaging, the most popular beamforming methods are based on the Delay-And-Sum technique (DAS). In synthetic aperture imaging techniques, this is based on selecting, from the acquisition dataset (Full Matrix Capture, FMC), the samples that correspond to the time-of-flight from the emitter at ${\vec{x}}_{i}$ to a point $\vec{\chi }$ in the Region of Interest (ROI) and back to the receiver at ${\vec{x}}_{j}$. The average of this selection of samples is an estimate of the ultrasonic reflection at $\vec{\chi }$. The extension of this operation to all the ROI points and all combinations of emitters and receivers is known as Total Focusing Method (TFM) [7]. This technique provides the maximum image quality that the aperture, with its natural apodization, can produce at each image point. The number of elements involved and the shape of their distribution in the coarray are important factors that limit the dynamic range and the lateral resolution attained.

**Figure 1:**
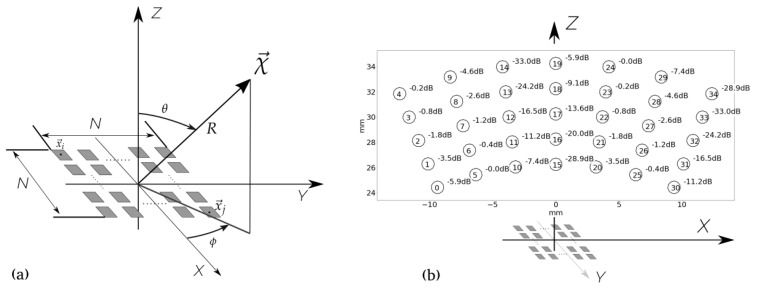
(**a**) Coordinate system used for beampattern simulation; (**b**) test scenario used to evaluate the imaging capabilities of the apertures.

In Figure 1a, a representation of a generic matrix array and the coordinate system used in this paper are illustrated. The full array is represented by a matrix structure of N×N elements. The number of parallel electronic channels operated in the aperture, in emission or reception, is defined as $N_{e}$, and, for simplicity in our discussion, $N_{e}$ will be considered the same in emission and reception. Consequently, this number coincides with the number of active aperture elements. When emission and reception apertures are different, the number of active elements can be up to $2N_{e}$. For a full array, $N_{e}=\left(N\times N\right)$, and when $N_{e}\ll \left(N\times N\right)$, we are working with a sparse array. We have selected as a reference full aperture a matrix of 20λ×20λ-size, composed of 40×40 elements (N=40) spaced by λ/2. In full aperture operation, $N_{e}=$ 1600.

Additionally, we simulated images in order to evaluate the response of the designed apertures in a more complex scenario than a single reflector. The scenario is illustrated in Figure 1b and is composed of 35 omnidirectional reflectors located in the near field with different reflectivities (from 0 dB to −33 dB) distributed in a sector (*R* [mm] = {25, 27, 29, 31, 33}, θ =$\{-22^{\circ }$, $-14.6^{\circ }$, $-7.3^{\circ }$, $0^{\circ }$, $7.4^{\circ }$, $14.6^{\circ }$, $22^{\circ }\}$). To emphasize the effect of the secondary lobes in the dynamic range, thermal or acoustic noise were not considered. Whilst all the beampatterns presented are computed with fixed focus in emission and reception, the images are computed using the FMC and the TFM.

For all simulations presented in the paper, $f_{c}=3.0$ MHz, BW = 60% and the propagation medium is water (propagation velocity c= 1500 m/s). Then, λ=c/f=0.5 mm and the full aperture is a 10 mm × 10 mm-square.

### 2.2. Delay-and-Sum Beamforming Technique

Considering a bidimensional array of $N_{e}$ elements, the subset of samples ($\mathrm{FMC}(\vec{\chi })$) used to evaluate the reflectivity at a point $\vec{\chi }$ of the image is defined as:

(1) $$\mathrm{FMC}\left(\vec{\chi }\right):\left\{s_{ij}\left(\vec{\chi }\right)=s_{ij}\left(\tau_{ij}\right),\;\tau_{ij}=\left|\frac{\vec{\chi }-{\vec{x}}_{i}}{c}\right|+\left|\frac{\vec{\chi }-{\vec{x}}_{j}}{c}\right|\;\forall i,j\in \left[1,N_{e}\right]\right\},$$

where ${\vec{x}}_{i}$ and ${\vec{x}}_{j}$ are the positions of the emission and the reception transducers, and $s_{ij}\left(\tau_{ij}\right)$ is the sample of the echo signal of the corresponding ij emission-reception pair at position $\vec{\chi }$. The echo signal is defined as:

(2) $$s_{ij}\left(\vec{\chi }\right)=m_{ij}\left(\vec{\chi }\right)+n_{ij}\left(\vec{\chi }\right)\approx m\left(\vec{\chi }\right)+n_{ij}\left(\vec{\chi }\right),$$

where $n_{ij}\left(\vec{\chi }\right)$ is composed of the contribution of all noise sources (acoustical, thermal, etc.), and $m_{ij}\left(\vec{\chi }\right)$ is the reflectivity value that depends on the particular response of the transducer pair and on the reflector diffraction response at $\vec{\chi }$. For convenience in this paper, all transducer elements and reflectors are considered omnidirectional and equal, so $m(\vec{\chi })$ is constant for all $s_{ij}\left(\vec{\chi }\right)$.

From the data contained in the $\mathrm{FMC}(\vec{\chi })$, the estimate of the reflectivity given by conventional DAS, $\hat{m}\left(\vec{x}\right)$, is given by:

(3) $$\hat{m}\left(\vec{\chi }\right)=\frac{1}{N_{e}^{2}}\sum_{i=1}^{N_{e}}\sum_{j=1}^{N_{e}}s_{ij}\left(\vec{\chi }\right)=m\left(\vec{\chi }\right)+\sum_{i=1}^{N_{e}}\sum_{j=1}^{N_{e}}\frac{n_{ij}\left(\vec{\chi }\right)}{N_{e}^{2}}=m\left(\vec{\chi }\right)+n\left(\vec{\chi }\right),$$

and $n(\vec{\chi })$ is the mean of all noise sources at $\vec{\chi }$.

### 2.3. Spatial Representation of Beamforming Information

The coarray is a well-known structure that models the behaviour of the pulse-echo response and is defined by the convolution of emission and reception apertures as:

(4) $$C\left(\vec{x}\right)=\sum_{i=1}^{N_{e}}f\left(\vec{x}-{\vec{x}}_{i}\right)*\sum_{j=1}^{N_{e}}f\left(\vec{x}-{\vec{x}}_{j}\right)=\sum_{i=1}^{N_{e}}\sum_{j=1}^{N_{e}}f\left(\vec{x}-\left({\vec{x}}_{i}+{\vec{x}}_{j}\right)\right),$$

where $f(\vec{x})$ is the element’s function that represents its spatial dimensions. Here we have used $f\left(\vec{x}\right)=\delta \left(\vec{x}\right)$, where δ(·) is the Dirac delta function, to simplify the analysis of the grid of the coarray element locations.

When we have the $\mathrm{FMC}(\vec{\chi })$, that corresponds to an image point $\vec{\chi }$, based on the emission-reception pairs, it is straightforward to introduce spatial information in the beamforming process by the projection of the samples in the coarray [29]:

(5) $$C_{s}\left(\vec{x},\vec{\chi }\right)=\sum_{i=1}^{N_{e}}\sum_{j=1}^{N_{e}}s_{ij}\left(\vec{\chi }\right)f\left(\vec{x}-\left({\vec{x}}_{i}+{\vec{x}}_{j}\right)\right).$$

**Figure 2:**
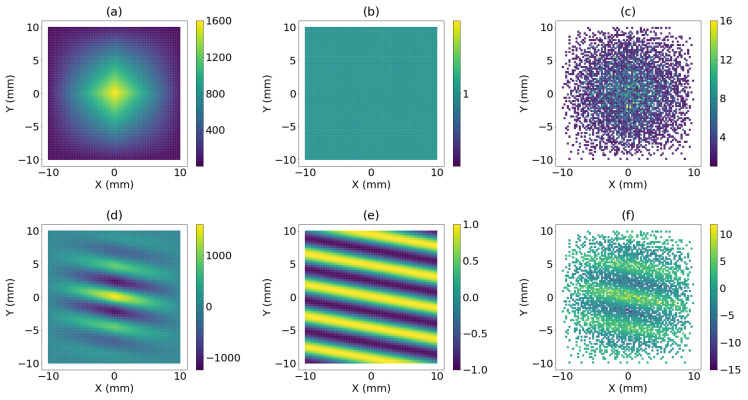
Projection of the $\mathrm{FMC}(\vec{\chi })$ when the reflector is located at ($R=100\;\mathrm{mm},\theta =0^{\circ },\phi =0^{\circ }$). If $\vec{\chi }=(R=100\;\mathrm{mm}$, $\theta =0^{\circ },\phi =0^{\circ })$ is in the reflector: (**a**) coarray of a full array; (**b**) coarray of minimum redundancy array; (**c**) coarray of sparse array. If $\vec{\chi }=\left(R=100\;\mathrm{mm},\theta =12^{\circ },\phi =80^{\circ }\right)$ is out of the reflector: (**d**) coarray of a full array; (**e**) coarray of minimum redundancy array; (**f**) coarray of sparse array.

This representation shows how data is spatially organized (see Figure 2). In the case of a 20λ-side aperture, we have simulated how the data $\mathrm{FMC}(\vec{\chi })$ is mapped in three different configurations: the full array, a flat coarray with one signal per element, and an example of sparse bin array with 100 elements. When the resultant coarray is composed of only one element per location, this is named Minimum Redundancy Coarray (MRC) [23,25,30]. When a reflector is at the focus point, in this case $\vec{\chi }=(R=100\;\mathrm{mm},\;\theta =0^{\circ }$, $\phi =0^{\circ })$ (see Figure 1 for coordinate system), the reflectivity is present in all signals, producing an increasing intensity in all coarray elements, as can be observed in Figure 2a–c.

When the source is out-of-focus, in this case $\vec{\chi }=(R=100\;\mathrm{mm},\;\theta =12^{\circ }$, $\phi =80^{\circ })$, it generates a wave front that falls obliquely in the coarray and draws a set of oscillation lines upon it. The relative position of the source to the focus determines the frequency of the oscillation pattern and its orientation. For the DAS beamforming process these oscillation patterns are acoustic noise that disturb the reflectivity estimation. In the case of the full array and sparse array the effect of the oscillation is smoothed by the natural apodization. In the case of the MRC, the oscillation generates extreme values in the beampattern and, in consequence, higher lateral resolution and higher sidelobes.

Based on the coarray, the estimate of the reflectivity can be obtained as:

(6) $$\hat{m}\left(\vec{\chi }\right)=\frac{{\;\int \int \;}_{\vec{x}}C_{s}\left(\vec{x},\vec{\chi }\right)d\vec{x}}{{\;\int \int \;}_{\vec{x}}C\left(\vec{x}\right)d\vec{x}}=\frac{{\;\int \int \;}_{\vec{x}}C_{s}\left(\vec{x},\vec{\chi }\right)d\vec{x}}{N_{e}^{2}},$$

and, for the beamforming process, the signals located at the same coarray position introduce the same information. Implicit in the DAS process, there is a well known spatial filtering operation generated by the coincidence of element distribution in the coarray. In the case of a full array, the natural apodization of its coarray corresponds to a pyramid-shape window. In the flat coarray, each position is filled with only one signal, and in sparse arrays, as the information is organized in an irregular distribution, side-lobe levels can be increased.

Based on these observations, it seems reasonable to propose solutions for sparse arrays based on the completeness of the coarray, and several works have addressed this objective in two different ways: searching two complementary apertures, one in emission and the other in reception, that generate this completeness [16,28]; alternatively, based on synthetic aperture, using different configurations in emission/reception to complete the coarray [30].

**Figure 3:**
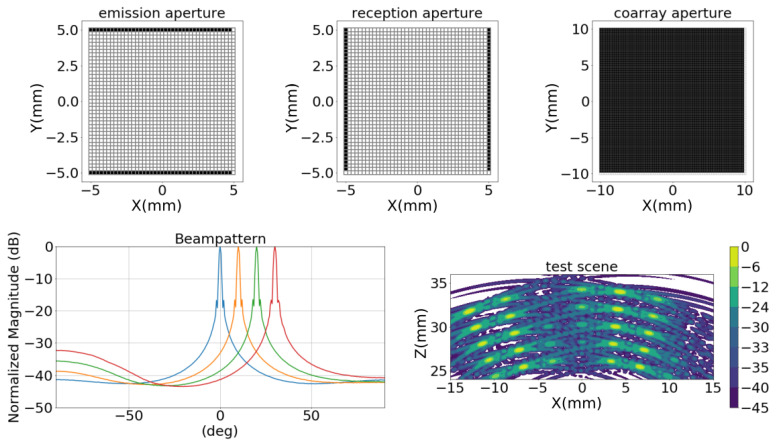
Sparse aperture solution to solve a minimum redundancy coarray. In the top, emission and reception apertures are presented with the resultant coarray. In the bottom-left, the beampattern in the *x*-axis (*R* = 100 mm, $\phi =0^{\circ }$,) is presented at different steering angles ( $0^{\circ }$ blue, $10^{\circ }$ yellow, $20^{\circ }$ green and $30^{\circ }$ red). In the bottom-right the image generated by this array from the scenario presented in Figure 1b.

An example of an MRC solution generated by two complementary apertures is presented in Figure 3. A flat coarray of (2N−1×2N−1) elements can be obtained by using two column arrays detached by (N+1) columns, and two row arrays detached by (N+1) rows. With $N_{e}=2N$ electronic channels and (4N−1) active elements in the aperture, this aperture fills the coarray with one signal per element. The beampatterns of the MRC (20λ-side), are also presented in Figure 3 for different steering conditions. The completeness in the coarray ensures that no grating lobes are generated, resulting in a high dynamic range of 41 dB, for $0^{\circ }$ steering. The beampattern also shows high lateral resolution and large sidelobes characteristics of flat arrays, and shows that, as the steering angle increases, the excitation of high-frequency components becomes more relevant, reducing the dynamic range. If the test scenario of Figure 1b is evaluated, complex sidelobe regions are generated, as can be observed in Figure 3. Although there is a good performance relative to lateral resolution, the structure generated by the sidelobes drastically decreases the dynamic range. At least 5 targets are not detected (13, 14, 15, 33 and 34), all of them with reflectivities under −24 dB.

The MRC generates a high lateral resolution that is maintained up to −18 dB, but sidelobes grow very fast, decreasing the lateral resolution and compromising the dynamic range. A way to avoid this is to increase the redundancy in the center of the coarray and generate at the same time a smooth coarray shape. This will decrease side lobe level but also diminish lateral resolution. Nevertheless, this side lobe suppression allows lateral resolution to be maintained throughout most of the dynamic range, with improved image and low-reflectivity target detection. Although the design of sparse random arrays is more complex [31], a random uniform distribution of the sparse elements produces a higher concentration of coarray elements in the center. Consequently, it seems to be more efficient than the MRC for imaging applications.

### 2.4. Sparse Binned Array and Sparseness Degree

A sparse binned array is divided into equal-sized bins (set of elements) and one element is chosen at random in each bin. With an uniform distribution of bins, it can be shown that the sparse binned array has the same properties of the sparse random array, except that the binned array has a sidelobe distribution that is much lower than the random array [26]. By its design, a binned array cannot generate the MRC, so it is an adequate example for our statistical metric analysis.

The first stage of sparse array design is to establish the potential to cover the coarray surface with a particular number of elements $N_{e}$. We have defined the concept of Sparseness Degree (SD) as the ratio between the number of positions to fulfill the coarray, $N_{c}={\left(2N-1\right)}^{2}$ (from a full matrix aperture of N×N), and the number of non-redundant signals that can ideally occupy different positions in the coarray, $N_{I}$:

(7) $$SD=\frac{N_{c}}{N_{I}}.$$

If the same aperture is used in emission and reception, then $N_{I}=\left(N_{e}^{2}-N_{e}\right)/2$, and if these apertures are absolutely different, then $N_{I}=N_{e}^{2}$.

When an aperture has a SD≥1, it is not possible to fulfill the area, and it is classified as an ultrasparse array. A value of SD<1 makes it easier to fill the coarray, but this is not guaranteed. We have considered that for a highly-sparse aperture, 0.5≤SD<1 (from one to two signals per coarray element), and for common sparse apertures, SD<0.5 (more than two signals per coarray element).

### 2.5. Beampattern Metric Analysis

**Figure 4:**
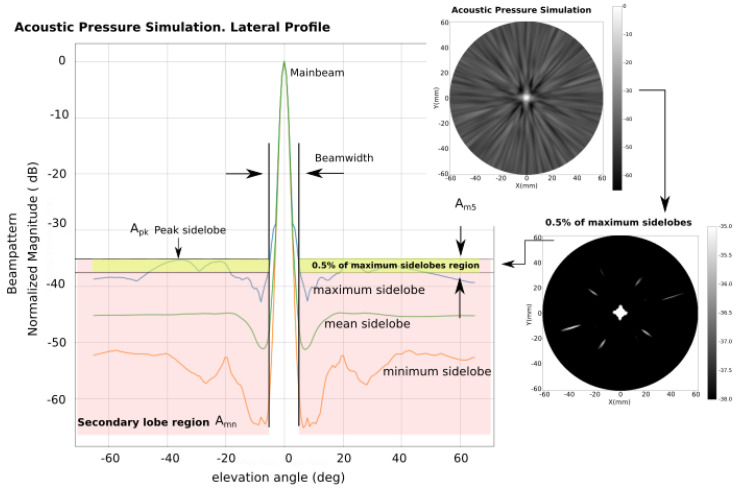
Elements used to evaluate the beampattern simulation. The beampattern is simulated in a semi-sphere ($\theta \in [-90^{\circ }:\Delta \alpha :+90^{\circ }]$, $\phi \in [0^{\circ }:\Delta \alpha :180^{\circ }]$, $\Delta \alpha =\frac{1}{2}\Delta \theta_{-6\mathrm{dB}}$). The three lateral profiles are composed of the semi-sphere by obtaining, at each elevation angle, the maximum, the mean and the minimum. The peak sidelobe $\left(A_{pk}\right)$ and the mainlobe width $\Delta \theta_{A_{pk}}$ are identified. The mean value of the sidelobes is $A_{mn}$. Furthermore, finally, the 0.5% percentile of the maximum sidelobes is identified and evaluated by its mean value ($A_{m5}$).

To evaluate the beampattern of a particular array, we have used the wideband Piwakovski solution with punctual elements to compute the acoustic pressure in the far-field semi-sphere [14]. Figure 4 illustrates the set of parameters used to evaluate the beampattern. The acoustic pressure is represented by the semi-sphere projection at each elevation angle ($\theta \in [-90^{\circ }:\Delta \alpha :+90^{\circ }]$, $\phi \in [0^{\circ }:\Delta \alpha :180^{\circ }]$, $\Delta \alpha =\frac{1}{2}\Delta \theta_{-6\mathrm{dB}}$). Then, the lateral profiles of the maximum, the mean and the minimum at each elevation angle are obtained.

Instead of using the classical −6 dB level to define the mainlobe width, we propose a new definition. In the lateral profile, the peak sidelobe $\left(A_{pk}\right)$ is identified, and from this level, the mainlobe width, $\Delta \theta_{A_{pk}}$, is determined. This definition is more adequate because it also represents a trade-off between dynamic range and lateral resolution. Once the mainlobe width is determined, from the sidelobe region we obtain the mean sidelobe value ($A_{mn}$), and the 0.5% percentile of the maximum sidelobes is identified and evaluated by its mean value ($A_{m5}$). All simulations have been obtained at steering angle of ($\theta =0^{\circ }$, $\phi =0^{\circ }$) with a Gaussian-enveloped sinusoidal pulse excitation at the frequency of 3 MHz (bandwidth of 60%). It is desirable to have low values of $A_{m5}$ and $A_{pk}$, which means better contrast, while a small value of $\Delta \theta_{A_{pk}}$ indicates good lateral resolution.

To determine the potential dynamic range resulting from a determined configuration, we have defined a threshold based on the one defined by Steinberg as the mean value of sidelobes in random apertures [20]. In our case, we have identified the number of non-redundant signals ($N_{I}$) that can ideally occupy different positions in the coarray, and considered the shadowing effects of the flat distribution projection in the principal axis as the worst case. Then, we have defined a threshold reference for $A_{m5}$ as:

(8) $$TH_{A_{m5}}=20\log\left(\frac{1}{\sqrt{N_{I}}}\right).$$

### 2.6. Coarray-Based Metric Analysis for Sparse Array Design

The sparse array design priority is to obtain a balanced coarray by maximizing the signal information regarding the imaged area, while covering the coarray surface as effectively as possible. In other words, a smooth natural apodization shape should be achieved. Based on these conditions, instead of using the beampattern as an optimization parameter, the design strategy focuses on balancing the statistics of the coarray redundancy distribution, that is represented by the weight of its elements.

The statistical parameters that are proposed in this work are: (i) the coarray occupied surface, $S_{a}$, evaluated as the proportion of occupied coarray positions; and two statistic parameters, related to the shape of the weights distribution $C(\vec{x})$: (ii) the variance $\left(\sigma^{2}\right)$ and (iii) the kurtosis (Ku). Our objective is to obtain a narrow distribution with a large surface value, so, whilst it is interesting to increase $S_{a}$, other objectives are to reduce both the variance and the kurtosis.

The coarray weight distribution is defined relative to $S_{a}$. Distributions with similar variance values are better rated if $S_{a}$ is higher. The kurtosis is related to the tails of the distribution [32]. The kurtosis of any univariate normal distribution is 3, and higher kurtosis corresponds to greater extremity of deviations, while lower kurtosis indicates that there are fewer and less extreme outliers when compared to the normal distribution. Here, we use the Karl-Pearson definition of kurtosis:

(9) $$Ku=E\left[{\left(\frac{C(\vec{x})-\mu }{\sigma }\right)}^{4}\right],$$

where *E* is the statistical expected value and μ is the mean value.

In essence, these parameters can only address the general behaviour of the coarray, so they are considered as low-pass filter evaluators. However, although we cannot guarantee the presence of high-frequency spikes in the sidelobe distribution, the low computational cost of these parameters allows developing a design process from a statistical perspective. To validate this interpretation, we have studied their relationship with the beampattern characteristics.

### 2.7. Incidence of Coarray Distribution Parameters in the Beampattern

In this work, we have analyzed the design problem of a 20λ-side sparse array. The corresponding full array would be composed of 40×40 elements spaced by λ/2. This aperture has, in the coarray, 6241 positions ($N_{c}$), and we have evaluated three configurations.

The first one, called 100I, contains 100 elements, operating all in emission and reception. In this case, $N_{I}=5050$ and SD=1.23, so it is considered an ultrasparse aperture. The aperture is divided in a grid of 10×10 bins and in each bin (4×4 elements), one element is active.

An alternative to decrease SD with the same number of parallel channels is to use different apertures in emission and reception. Thus, the second configuration, called 100V, contains 100 elements in emission and 100 in reception. No conditions about overlapping were supposed. In this case, $N_{I}=10,000$ and SD=0.62, and we classify it as a high-sparse aperture. The aperture is divided in a grid of 10×10 bins and in each bin (4×4 elements), two elements are active, being one in emission and other in reception.

The third configuration, called 196I, is composed of 192 elements operating all in emission/reception. In this case, the aperture is 21λ wide, $N_{I}=19,304$ and SD=0.32, resulting in a sparse aperture. The aperture is divided in a 14×14 grid and at each bin (3×3 elements) one element is active.

For each configuration, 100,000 cases of bin arrays were randomly generated. For each case, the coarray was computed and the values of $S_{a}$, $\sigma^{2}$ and $Ku\left[C(\vec{x})\right]$ were calculated. Furthermore, the beampattern was computed and summarized using the four values previously defined: the average of side-lobe region ($A_{mn}$), peak side-lobe ($A_{pk}$), the mean of the percentile 0.5% of maximum side-lobes ($A_{m5}$) and the mainlobe width ($\Delta \theta_{A_{pk}}$). Additionally, the threshold value $TH_{A_{m5}}$ has been used to classify the apertures in two sets. Then, the distribution of both sets has been evaluated along the $S_{a}$, $\sigma^{2}$ and $Ku\left[C(\vec{x})\right]$ axis, and are presented with the distribution of cases at each value.

**Figure 5:**
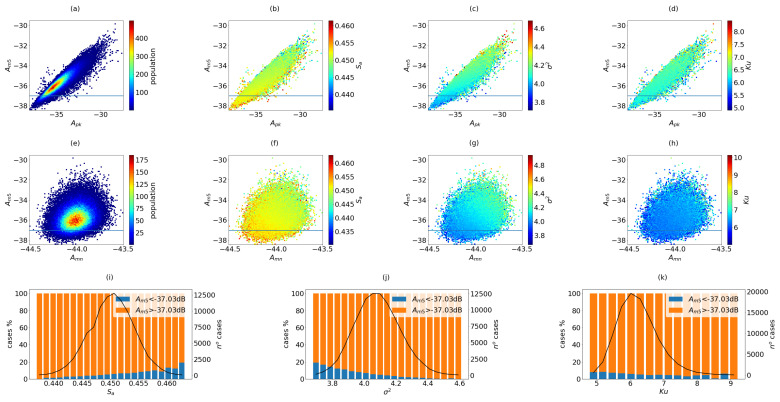
Results of 100,000 random cases of configuration 100I. In the first row, the apertures are presented in a $A_{m5}$ vs. $A_{pk}$ map, (**a**) population, (**b**) $S_{a}$, (**c**) $\sigma^{2}$ and (**d**) Ku. In the second row, the apertures are presented in $A_{m5}$ vs. $A_{mn}$ map, (**e**) population, (**f**) $S_{a}$, (**g**) $\sigma^{2}$ and (**h**) ku. In the third row, the distribution of cases against the coarray parameters are presented, marking the proportion of cases that fulfill the $TH_{A_{m5}}$ threshold, (**i**) $S_{a}$, (**j**) $\sigma^{2}$ and (**k**) Ku.

**Figure 6:**
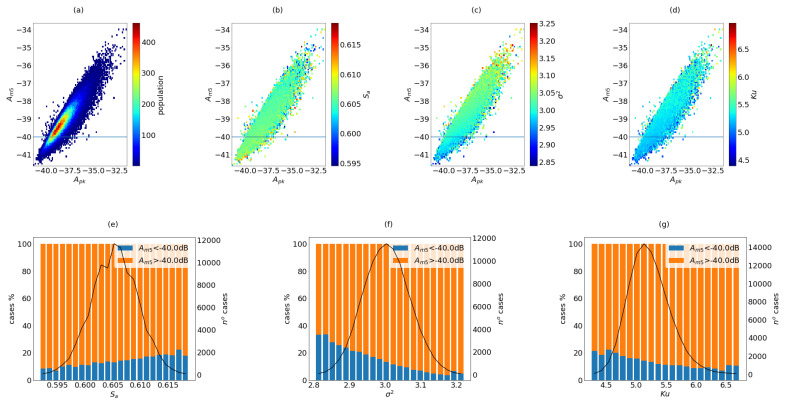
Results of 100,000 random cases of configuration 100V. In the first row, the apertures are presented in $A_{m5}$ vs. $A_{pk}$ map, (**a**) population, (**b**) $S_{a}$, (**c**) $\sigma^{2}$ and (**d**) Ku. In the second row, the distribution of cases against the coarray parameters are presented, marking the proportion of case that fulfill the $TH_{A_{m5}}$ threshold, (**e**) $S_{a}$, (**f**) $\sigma^{2}$ and (**g**) Ku.

**Figure 7:**
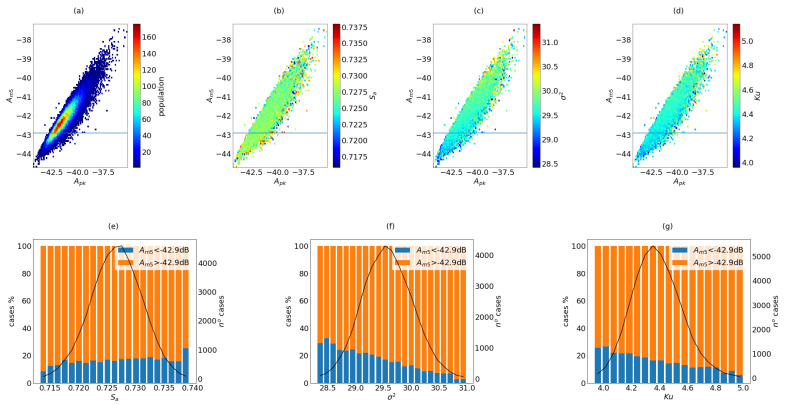
Results of 50,000 random cases of configuration 196I. In the first row, the apertures are presented in $A_{m5}$ vs. $A_{pk}$ map, (**a**) population, (**b**) $S_{a}$, (**c**) $\sigma^{2}$ and (**d**) Ku. In the second row, the distribution of cases against the coarray parameters are presented, marking the proportion of case that fulfill the $TH_{A_{m5}}$ threshold, (**e**) $S_{a}$, (**f**) $\sigma^{2}$ and (**g**) Ku.

The results of the simulations per configuration are summarized in Figure 5, Figure 6 and Figure 7. These plots are useful to observe how the coarray parameters are related to the beampattern parameters.

#### 2.7.1. The 100I Configuration

This case is classified as an ultrasparse aperture (SD=1.23), and the results from the 100,000 simulations are presented in Figure 5.

Figure 5a–d show the population distribution, $S_{a}$, $\sigma^{2}$ and Ku, plotted as a function of $A_{m5}\times A_{pk}$ in dB, respectively. The results are presented in scatter plots obtained from the mean of five neighbour samples, and are useful to observe how the coarray parameters are related to the beampattern parameters. It is desirable to have low $A_{m5}$ and $A_{pk}$, which means better contrast. The threshold $TH_{A_{m5}}$ is represented by the horizontal line, whereas the good apertures should exhibit parameters below the threshold. For this configuration, the occupancy of the coarray is around 45%.

The values of $A_{pk}$ are in the range from −37 to −31 dB, and the values of $A_{mn}$ were concentrated in a small window, smaller than 0.5 dB around the value of −43.5 dB, showing a weak relevance of this parameter to evaluate the beampattern, so it was not presented in the results.

In Figure 5a, the population distribution can be observed as a function of the sidelobe characteristics. In Figure 5b, it is possible to identify results with higher occupancy and lower sidelobes. Lower values of variance (Figure 5c) can be also correlated with lower sidelobes, and it can be observed that higher values of kurtosis (Figure 5d), larger than 6, means that there is a significant number of outliers, but there are also solutions with low kurtosis and lower sidelobes.

In the second row, Figure 5e–g, the threshold $TH_{A_{m5}}=-37.03$ dB has been applied to classify the solutions, showing the proportional distributions of $S_{a}$, $\sigma^{2}$ and Ku, respectively. The blue and orange bars represent the proportion of apertures that generate beampatterns whose $A_{m5}$ value are less and greater than the threshold value $TH_{A_{m5}}$, respectively, and the black line represents the distribution of binned arrays related to the calculated statistical parameters. From this value, the $A_{pk}$ value can oscillate in a range of 2.5 dB. It can be seen that the presence of good apertures increases with low values of variance and high area (up to 20%). Although it seems to be a trend that the number of good apertures increases for low kurtosis values, it has less significance.

We can conclude that there is a correlation between these parameters and the level of sidelobe distribution, that is, the apertures with large occupancy, low variance or low kurtosis are more frequent in the region where secondary lobes are lower.

#### 2.7.2. The 100V Configuration

Using different apertures for emission and reception (non-overlap conditions were applied) doubles the value of $N_{I}$ and increases the coarray occupancy a 20% ($S_{a}$ is around 0.605). The results are shown in Figure 6. The population and the distribution of the coarray parameters show a similar distribution when compared with the ultrasparse case. However, there is an increment in the range of the beampattern parameters. In this case, the dynamic range rises to −40.5 dB ($A_{m5}$), while the $A_{mn}$ parameter shows a stable value around −46.5 dB, with variation smaller than 0.35 dB (not shown in the figure).

The variance is now around 3 and the kurtosis, although it still reports the presence of outliers, has a maximum of 5.8 and shows a clearer influence. If the evolution of $A_{m5}$ is considered, it seems that, for $S_{a}$, the proportion of better cases is stagnant at 20%. However, it increases with Ku and $\sigma^{2}$, almost doubling the proportion of good apertures.

#### 2.7.3. The 196I Configuration

This configuration almost doubles the number of transducers and of electronic channels. For our convenience, for this configuration the number of random apertures has been reduced to 50,000, and the results are shown in Figure 7. In this case, although the value of $N_{I}$ is 3.8 times higher than the number of coarray locations, the value of $S_{a}$ reaches only 74%. The population and the distribution of the coarray parameters show a similar distribution than in the other cases. However, in this case, the dynamic range rises to −43.5 dB, while $A_{mn}$ shows a stable value around −50 dB, with variation smaller than 0.5 dB (not shown in the figure).

For this configuration, it seems that the dynamic range is more related to the shape of the coarray than to the $S_{a}$ value. Although the variance shows higher values than in the previous configurations, the kurtosis is lower, but it still reports outliers. If the distribution of good apertures ($A_{m5}<42.9$ dB) is studied, the results show that the beampattern is improved when the variance and the kurtosis are reduced, showing the same trends as in the previous configurations.

## 3. Fitness Functions

In this section, the fitness functions are proposed, evaluated and tested by using the coarray and beampattern parameters, and simulated scenarios.

### 3.1. Coarray-Based Fitness Function

From Figure 5, Figure 6 and Figure 7, we can identify a set of coarray features that can be used as the basis of our design methodology. Although it could be concluded that the most significant coarray parameter is the variance, this criterion is useful if a large surface and a low kurtosis are achieved. These parameters evolve in different scales, so the fitness function is defined in a proportional form to compare two different apertures *n* and *m*. Furthermore, to avoid a local minimum, this function has been defined as a balanced factor between the three parameters:

(10) $$FF\left[n,m\right]=\frac{\sigma^{2}\left[n\right]}{\sigma^{2}\left[m\right]}+\frac{Ku[n]}{Ku[m]}+\frac{S_{a}\left[m\right]}{S_{a}\left[n\right]}.$$

The computational cost of this figure of merit is very low, and we can compare thousands of solutions very rapidly. One candidate aperture, say *m*, is evaluated, and after that, a random number of bins in the *m*-aperture are mutated. The new aperture, say *n*, is analyzed against the previous candidate. If FF[n,m]<3, the *n*-aperture is considered better than the *m*-aperture, and it will occupy the place of best aperture candidate.

Due to the nature of the binned array, we have eliminated the possibility of evolution to an MRC. However, the coarray that this distribution produces can be very dispersive and irregular. Using the proposed fitness function we try to reduce high concentration of redundant signals and reduce the number of empty positions. This smooth distribution will keep the spatial resolution, while reducing secondary and grating lobes.

To evaluate this function, we have created a very simple search algorithm, and the process is initiated with a random candidate aperture. The parameters are calculated, then the aperture is mutated and evaluated against the previous configuration. If the new aperture is better than the previous one, the mutated aperture is the new candidate and the process is repeated. The number of mutated bins is a random value between one and a value represented by 5% of bins. The algorithm finishes when 20,000 apertures are tested without a new candidate.

**Figure 8:**
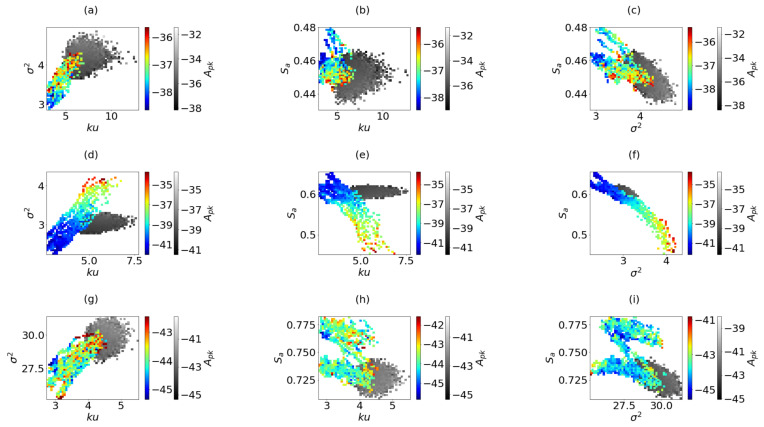
In color, the values of $A_{m5}$ reached by the candidates apertures in the search process. In gray, the values of $A_{m5}$ obtained with 100,000 random apertures. Configuration 100I: (**a**), $\sigma^{2}\times Ku$, (**b**), $S_{a}\times Ku$, and (**c**) $S_{a}\times \sigma^{2}$. Configuration 100V: (**d**), $\sigma^{2}\times Ku$, (**e**), $S_{a}\times Ku$, and (**f**) $S_{a}\times \sigma^{2}$. Configuration 196I: (**g**), $\sigma^{2}\times Ku$, (**h**), $S_{a}\times Ku$, and (**i**) $S_{a}\times \sigma^{2}$ for configuration 196I.

In order to evaluate the performance of the new fitness function, the algorithm was executed sixteen times per configuration in independent searches. At each search, when the new *n*-aperture surpasses the fitness function, the beampattern is evaluated to annotate the evolution of the resolution and the dynamic range ($A_{m5}$, $A_{mn}$ and $A_{pk}$). In total, including the three configurations and all the searches, 4,543,575 apertures were evaluated and only 6230 were candidate apertures. The results are presented in Figure 8. The 6230 solutions are organized by configuration, and presented in different maps referenced by the coarray parameters, where $A_{m5}$ is represented in color. Furthermore, the results of the previous random simulations are presented in gray colormap as reference. Results in Figure 8 are organized as $\sigma^{2}\times Ku$, $S_{a}\times Ku$ and $S_{a}\times \sigma^{2}$ (columns) for configurations 100I, 100V and 196I (rows).

The results show that, as expected, better results are produced with low variance, low kurtosis and larger area. The new results are in regions where the random apertures were not able to reach, which indicates that the search algorithm converges to better solutions. The dynamic ranges, measured as of $A_{m5}$, are slightly superior to those obtained by the random binned apertures. However, the proportion of good cases is superior and they are located in specific areas. This last asseveration is clear in the case of 100V, where we can identify these regions in the three representations of Figure 8d–f. Furthermore, in 100I, where the number of better apertures is smaller. Consequently, it is easy to identify a search area where to obtain a sparse aperture. However, in the case of 196I, the behaviour is different for each representation and it is difficult to identify a specific area for searching. It is important to remark that these results are a consequence of an evolutionary process, and, for the case 100V, this evolution from bad apertures, out of the random set, to good apertures, is very straight. However, in the other two apertures this evolution is very chaotic. In particular in 196I, the evolution of the algorithm does not always converge to good apertures. This is better shown if the beampattern evolution is analyzed.

#### 3.1.1. Evolution

**Figure 9:**
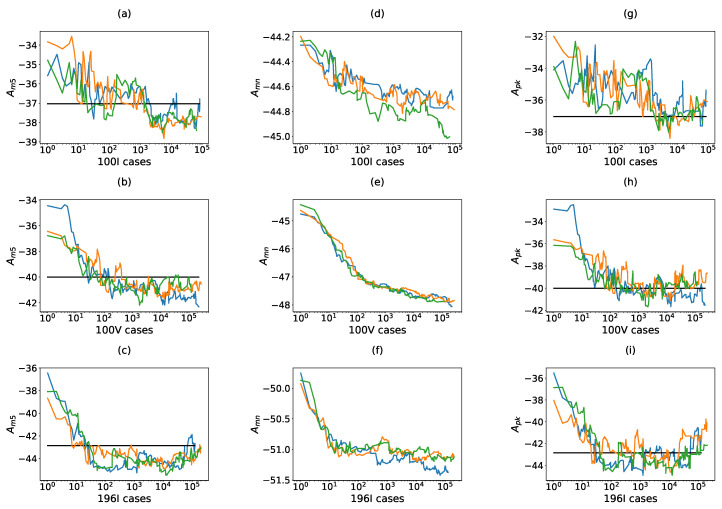
Coarray-based fitness function. Each row analyses the evolution of the dynamic range in one specific configuration. For 100I: (**a**) $A_{m5}$, (**d**) $A_{mn}$, (**g**) $A_{pk}$. For 100V: (**b**) $A_{m5}$, (**e**) $A_{mn}$, (**h**) $A_{pk}$. For 196I: (**c**) $A_{m5}$, (**f**) $A_{mn}$, (**i**) $A_{pk}$. For each configuration three different evolution processes are presented (colors blue, orange and green). In the figures where $A_{pk}$ or $A_{m5}$ are presented, the threshold line $TH_{A_{m5}}$ has been indicated.

Figure 9 illustrates the evolution of better results found for three searches per configuration, where $A_{m5}$, $A_{mn}$ and $A_{pk}$ (columns) are analyzed for the 100I, 100V and 196I configurations (rows). In the figures corresponding to $A_{m5}$ and $A_{pk}$, a horizontal line indicates the threshold level $TH_{A_{m5}}$.

The first observation is centred on the fact that all configurations converge to higher dynamic range solutions. In the case of configuration 100I (first row), this convergence is weaker and noisier for all the parameters, mainly at the beginning of cases. However, for 100V and 196I (second and third lines) the convergence for $A_{m5}$ is faster and stabilized when $TH_{A_{m5}}$ is reached. For $A_{pk}$ there is also a fast convergence for the first cases, but at the end it tends to be situated around the threshold level.

In general, it is not a complex issue to achieve the $TH_{A_{m5}}$ threshold, and, although for the ultrasparse configuration several thousand evaluations are needed, in high sparse and sparse apertures this threshold is achieved in a few hundred cases.

The computational cost of the process can be evaluated from Table 1, which presents, for each configuration, the number of apertures evaluated (by the coarray parameters) and the number of candidate apertures, for the three searches. The number of apertures evaluated for each case is large, but this is not an unusual number [17]. Nonetheless, the computational cost is very reduced when compared to a conventional fitness function. The difference in the number of evaluations needed to generate a solution between 100I and 100V is due to the fact that in this last case the mutation is applied in both apertures (emission and reception).

#### 3.1.2. Results and Discussion

The simplicity of the optimization process, without an explicit mechanism to avoid process stagnation, has led us to consider the design from a statistical perspective. All solutions that exceed the threshold value are considered viable (or good) solutions. When the search algorithm finishes, the set of viable solutions is studied to select the most suitable aperture.

In Table 2, the beampattern parameters for each solution are presented. If they are compared with the results of random apertures, all the results are in the top, or, as in the case of 196I, over the top. The more remarkable result is that using the same number of electronic channels, employing different apertures for emission and reception can improve the results with no significant increase in resources and complexity. If the number of electronic channels is doubled, meaning to pass from an ultrasparse aperture to a sparse aperture, the dynamic range can be increased by almost 8 dB.

**Figure 10:**
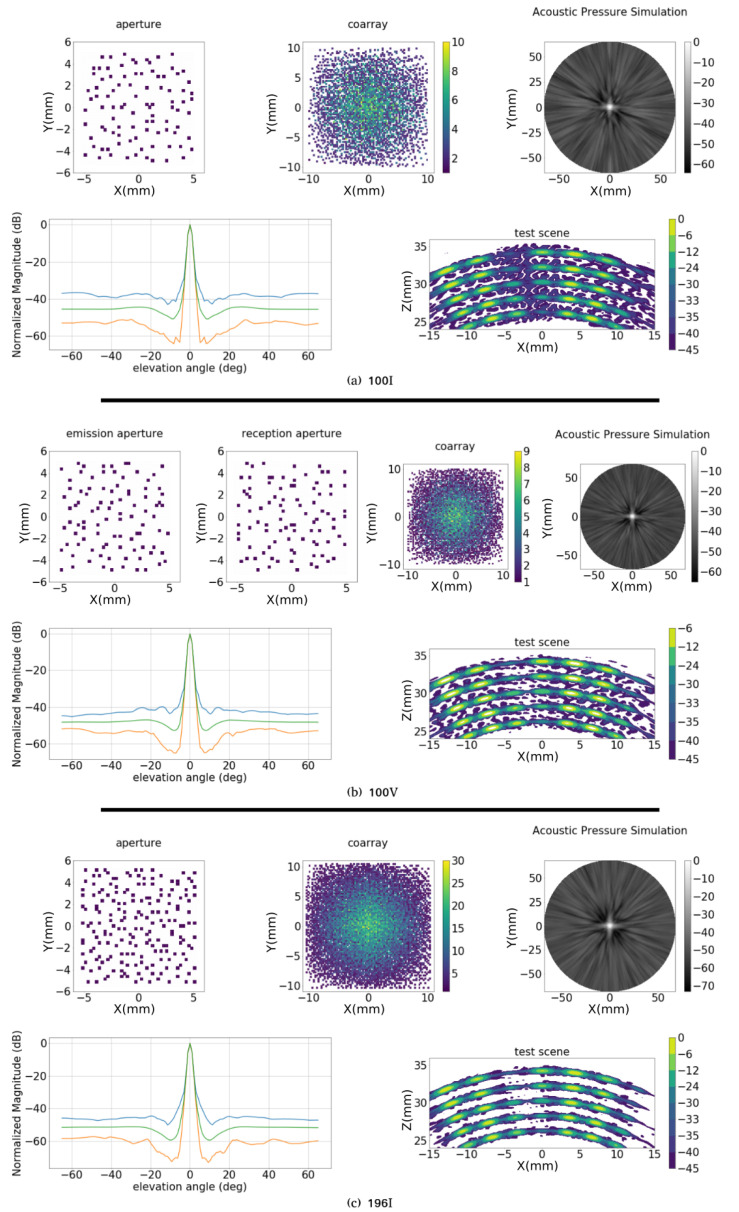
Optimization with coarray based fitness function. Best results for configurations (**a**) 100I, (**b**) 100V and (**c**) 196I. In each figure are presented: emission (and reception for 100V) apertures; coarray matrix representation; acoustic field in the semi-sphere; maximum (blue), mean (green) and minimum (red) lateral profile at each elevation angle; and image resulting from the test scenario.

In Figure 10a–c, the best solutions for the three configurations are presented. In contrast with the results obtained for the MRC, the images in Figure 10 of the test scenario are more consistent with the corresponding dynamic range of the aperture. Although the MRC shows better lateral resolution in the range up to −12 dB, starting from this level all the binned arrays produce better results. In the case of 100I, in spite of its dynamic range being worse than the MRC’s, it can resolve all the targets. In aperture 100V (Figure 10b), the resulting apertures share 6 elements, which means that the number of active elements is 194.

### 3.2. Combined Fitness Function

Despite the simplicity of the proposed process, it is possible to improve the convergence of the optimization process if the evolution step is also driven by the results of the beampattern and we combine coarray parameters and beampattern into one fitness function. Now, once a candidate is selected according to the coarray fitness function (FF[n,m]<3), the beampattern is evaluated, and the new candidate is promoted if the condition $A_{m5}\left[n\right]<A_{m5}\left[m\right]$ is achieved. The choice for $A_{m5}$ is intended to reduce even more the sidelobe levels. The stop condition is also reduced: if after 3000 mutations the coarray fitness function cannot produce a solution, the process is finished.

#### 3.2.1. Evolution

**Figure 11:**
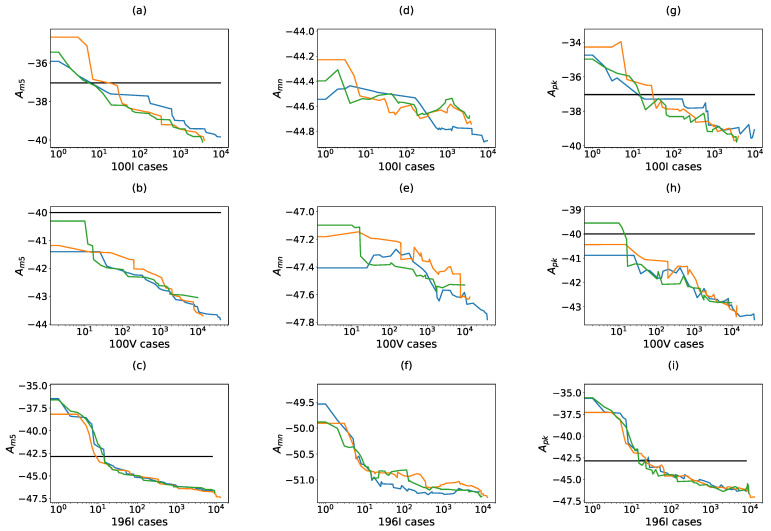
Combined fitness function. Each row analyses the evolution of the dynamic range in one specific configuration. For 100I: (**a**) $A_{m5}$, (**d**) $A_{mn}$, (**g**) $A_{pk}$. For 100V: (**b**) $A_{m5}$, (**e**) $A_{mn}$, (**h**) $A_{pk}$. For 196I: (**c**) $A_{m5}$, (**f**) $A_{mn}$, (**i**) $A_{pk}$. For each configuration three different evolution processes are presented (colors blue, orange and green). In the figures where $A_{pk}$ or $A_{m5}$ are presented, the threshold line $TH_{A_{m5}}$ has been indicated.

The evolution of the beampattern with this fitness function is presented in Figure 11, where three design processes are presented for each configuration. The results reveal that we achieve better dynamic range in all configurations when compared to the cases that employed the coarray-based fitness function. Furthermore, the convergence to a solution is produced faster. From the starting aperture, the improvements in $A_{m5}$ can go up to 10 dB (196I). It is interesting to remark that $A_{pk}$ evolves in a similar way in all the cases, reducing the oscillation that was characteristic in the first proposition. However, the value of $A_{mn}$ evolves slowly and, as opposed to Figure 9, the search lines of apertures 100I and 100V stop abruptly. On the other side, in 196I the search lines show a convergence to the solution. In the case of 100V the three random apertures that initialized each optimization algorithm are already below the threshold (see Figure 11b). This finding is perfectly compatible with the results of Figure 6.

Concerning the computational cost, several examples are summarized in Table 3, where the number of the apertures evaluated, the beampatterns computed and the number of candidates produced at each configuration, have been annotated. Although the number of beampatterns is significant, the convergence is faster and the number of tested apertures has been highly reduced. These numbers are in correspondence with the naive design of the implemented optimization algorithm and inside each configuration there is a high variability.

Finally, we have to indicate the computational cost of evaluating the beampattern is 58.55 times higher than the computation cost for evaluation of the coarray parameters.

#### 3.2.2. Results and Discussion

**Figure 12:**
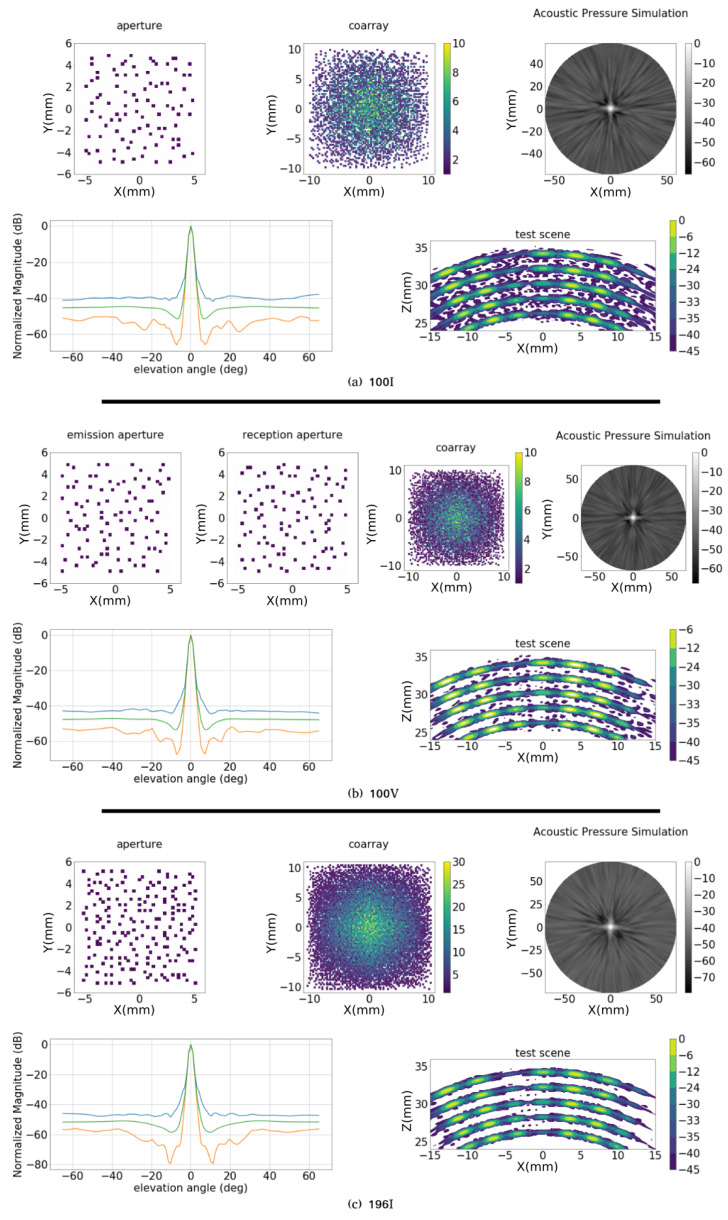
Optimization with combined fitness function. Best results for configurations (**a**) 100I, (**b**) 100V and (**c**) 196I. In each figure are presented: emission (and reception for 100V) apertures; coarray matrix representation; acoustic field in the semi-sphere; and, maximum (blue), mean (green) and minimum (red) lateral profile at each elevation angle; and image resulting from the test scenario.

If the combined Fitness Function is used, the results are improved. The best solutions obtained for each configuration are presented in Figure 12 and in Table 4. Considering the apertures obtained in the previous section, the $A_{mn}$ values are the same, but there is an improvement in the dynamic range (2 to 3 dB). For the aperture 100V the dynamic range reaches 42 dB, while for 196I, it is 47 dB. The bandwidth in both sets are similar, and an interesting point is that, for the solutions obtained by the combined Fitness Function, the differences between $A_{m5}$ and $A_{pk}$ are smaller.

When compared with the results obtained in Figure 10, the images of the test scenario in Figure 12 show a reduction in the sidelobe distribution, specially for the apertures 100I and 100V. In Figure 12a, the test scene has a large sidelobe region from −35 dB and all the targets are identified. In Figure 12b, aperture 100V, the secondary lobes are reduced to the −40 dB region. For this solution the overlap between emission and reception are 33 elements, so the final number of active elements is 167. In Figure 12c, aperture 196I, the result is similar to Figure 10c. However, some repetitive structures are generated between the arcs under the range of −40 dB.

Although the literature has plenty of examples of sparse apertures, the diversity in the cases turns it difficult to obtain a fair comparison between different works. Just to show a comparison, in [16] the authors report several sparse matrix arrays with a vernier-like configuration (13.5λ-diameter). Their results, using 140 elements in emission and 61 in reception, achieve a 35 dB dynamic range; with 135 elements in reception, it achieves a dynamic range of 44 dB; and with 245 elements in reception, it achieves a dynamic range of 44 dB. In this sense, we can conclude that the coarray parameters are useful to address the sparse array design problem and can obtain results comparable to other design strategies.

## 4. Conclusions

This paper examines the potential of using the distribution of weights in the coarray to characterize the beampattern of the aperture. Three configurations with different levels of sparseness have been studied: ultrasparse, high sparse and sparse. Furthermore, we have defined a threshold level for the dynamic range, depending on the number of non-redundant signals involved in the beamforming process. To avoid the inconvenience of a flat coarray, we have centred our analysis on matrix sparse binned arrays, so the conclusions are related to this particular configuration. We have shown that there is a relationship between the dynamic range and the parameters of the distribution of weights in the coarray. The variance, the kurtosis and the covered area are significant to define a set of apertures that reach or even exceed the threshold. This leads to apertures with corresponding coarrays of large areas, low variance and low kurtosis.

On the basis of these coarray parameters, we have defined a fitness function of low computational cost that has been used to design the three types of aperture. However, these parameters work like a low-pass filter and they cannot report outliers in the form of isolated high sidelobes that can reduce the dynamic range. So, due to the low cost of the fitness function, a statistical approach was followed to find the best solution.

The results show that the fitness function is effective to evaluate all the proposed configurations, where the best performance is attained for high sparse apertures. However, once the threshold is satisfied, the evolution addressed by the coarray-based fitness function produces oscillations in the dynamic range. Using the coarray parameters and $A_{m5}$, a combined fitness function is proposed to eliminate this behaviour. In this case, only when the coarray-based fitness function is attained, the beampattern is computed. Although the computational cost increases because of the beampattern simulation, the number of studied apertures is significantly reduced, and the final results are better than when using only the coarray evaluation.

As an example, a 20λ-side sparse aperture has been designed with a different number of elements. The results show that with one hundred elements and the same aperture in emission and reception, the dynamic range is slightly under 40 dB. In the case of using different apertures with the same number of electronic channels, the dynamic range reaches 42 dB. When the number of electronic channels and transducers are doubled, the dynamic range can rise to 47 dB. Furthermore, we have defined a complex scenario to test the imaging capabilities of the binned array solutions and compared them with the MRC. The beampatterns of all the apertures indicate a dynamic range around 40 dB. However, the MRC can only resolve targets under −24 dB. On the other hand, all the designed binned arrays can resolve the test image with a sidelobe distribution around 5 dB over its theoretical dynamic range.

Future works will be addressed to develop a similar analysis for non-grid apertures. Furthermore, we are interested in integrating the combined fitness function as a balanced multi-objective fitness function, in a more sophisticated optimization algorithm. Finally, we are interested in conducting a comparative analysis of the performance of apertures with similar dynamic range but different coarray statistical parameters.

## Figures and Tables

**Table 1:** Computational cost of the each optimization search line (apertures evaluated: candidate apertures).

Configuration	Search 1	Search 2	Search 3
100I	84,160:96	92,204:117	65,828:87
100V	222,104:169	266,043: 173	119,884:149
196I	125,333:160	197,296:175	202,446:161

**Table 2:** Summary of the beampattern parameters for the best solutions at each configuration. Coarray-based fitness function).

Configuration	$A_{m5}$ [dB]	$A_{\mathrm{mn}}$ [dB]	$A_{\mathrm{pk}}$ [dB]	$\Delta \theta_{A_{\mathrm{pk}}}$
100I	−37.32	−44.94	−36.66	$13.33^{\circ }$
100V	−41.56	−47.64	−40.12	$16.07^{\circ }$
196I	−45.14	−51.31	−44.03	$16.07^{\circ }$

**Table 3:** Computational cost for different configurations. Combined fitness function.

Configuration	Apertures	Beampatterns	Candidates
100I	6380	209	16
100I	5620	292	17
100V	13,436	491	33
100V	9788	858	23
196I	10,892	517	41
196I	12,761	1039	49

**Table 4:** Summary of the beampattern parameters for the best solutions at each configuration. Combined fitness function).

Configuration	$A_{m5}$	$A_{\mathrm{mn}}$	$A_{\mathrm{pk}}$	$\Delta \theta_{A_{\mathrm{pk}}}$
100I	−40.00	−44.80	−39.45	$13.84^{\circ }$
100V	−42.88	−47.44	−42.25	$12.85^{\circ }$
196I	−47.25	−51.37	−47.03	$18.62^{\circ }$
